# Building evidence on safety of endovascular thrombectomy for patients under anticoagulation with vitamin K antagonists

**DOI:** 10.1111/cns.14777

**Published:** 2024-07-03

**Authors:** Li Gao, Xiaowei Sun, Peiying Li

**Affiliations:** ^1^ Department of Neurology, Ren Ji Hospital Shanghai Jiao Tong University School of Medicine Shanghai China; ^2^ Clinical Research Center, Ren Ji Hospital Shanghai Jiao Tong University School of Medicine Shanghai China; ^3^ Department of Anesthesiology, Ren Ji Hospital Shanghai Jiao Tong University School of Medicine Shanghai China

**Keywords:** anticoagulation, endovascular thrombectomy, ischemic stroke, symptomatic intracranial hemorrhage, vitamin K antagonists

## Abstract

A recent study by Brian Mac Grory and colleagues investigated the safety of endovascular thrombectomy (EVT) among patients under vitamin K antagonists (VKAs) use within 7 days prior to hospital admission. Through this retrospective, observational cohort study, they found prior VKA use did not increase the risk of symptomatic intracranial hemorrhage (sICH) overall. However, recent VKA use with a presenting international normalized ratio (INR) > 1.7 was associated with a significantly increased risk of sICH. Future large‐scale randomized controlled trials should be conducted to further clarify the effects and feasibility of EVT therapy in ischemic stroke patients under anticoagulation.

## Graphical abstract

In this study, a total of 32,715 patients with acute ischemic stroke undergoing endovascular thrombectomy (EVT) were included. In total, 29,628 patients (90.6%) were not taking a vitamin K antagonist (VKA) prior to stroke, and 3087 patients (9.4%) were taking a VKA. The incidence of symptomatic intracranial hemorrhage (sICH) among the VKA group is 6.8%, which is comparable to those non‐VKA users with an incidence of 6.4%. Then, the international normalized ratio (INR) was dichotomized as ≤1.7 and >1.7 to assess the risk of sICH in each subgroup. Among 830 patients taking a VKA with an INR greater than 1.7, the incidence of sICH was 8.3%, which is significantly higher than those not taking a VKA (6.4%). Meanwhile, those with an INR of 1.7 or lower (*n* = 1585) had no significant difference of the risk of sICH (6.7%).

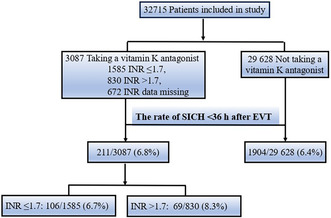

Acute ischemic stroke (AIS) is the major cause of death and disability worldwide. Timely reperfusion with intravenous thrombolysis (IVT) or endovascular thrombectomy (EVT) is currently the most effective way to improve neurological outcomes of AIS patients, especially those with large vessel occlusion. However, reperfusion with IVT is contraindicated in patients taking direct oral anticoagulants (DOACs) or vitamin K antagonists (VKAs) with an elevated international normalized ratio (INR) > 1.7. The benefit of EVT in those anticoagulated patients is not yet fully established due to the increased risk of symptomatic intracranial hemorrhage (sICH). Although some previous studies examined the association between VKA use and the risk of sICH in AIS patients undergoing EVT, the results are conflicting.^1^, ^2^, ^3^, ^4^, ^5^, ^6^, ^7^ Therefore, exploring the safety of EVT in the context of VKA use in clinical practice is of great importance.

In the issue of JAMA on June 20, 2023, Brian Mac Grory and colleagues^8^ investigated the association between VKA use within the 7 days preceding hospital admission and the occurrence of sICH within 36 h among 32,715 patients undergoing EVT in 594 hospitals. Overlap weighting, a propensity score weighting method, was employed to achieve a precise balance in covariates between patients who were either taking or not taking a VKA. After risk adjustment, recent VKA use within 7 days prior to EVT was not significantly associated with increased risk of sICH. However, the risk of sICH was increased among the VKA‐treated patients when an INR>1.7 at the time of admission. Despite the higher risk of sICH in patients with an elevated INR, there were no statistically significant differences with respect to in‐hospital mortality, the ability to ambulate at discharge, functional independence, or freedom from disability after risk adjustment between the VKA and non‐VKA users.

This retrospective, observational cohort study specifically addresses the safety of EVT among patients taking VKAs within 7 days prior to hospital admission. The incidence of sICH among the VKA group is 6.8%, which is comparable to those non‐VKA users with an incidence of 6.4%, providing supportive data for clinicians who plan to perform EVT for patients with recent VKA use. Then, the investigators dichotomized INR as ≤1.7 and >1.7 to assess the risk of sICH in each subgroup among non‐VKA users. They also explored the association between VKA and receipt of intravenous tPA on the endpoints. Further, separate logistic regression models were created to examine the adjusted association between a continuous measure of INR and each binary endpoint. The main strength of the study is that it includes a large number of such patients across diverse institutions with heterogeneous standard practices for EVT with recent VKA use.

The clinical implications of these findings are of great importance for the large population of AIS patients who could benefit from EVT when reperfusion is not achieved with tPA or with exclusion criteria for IVT. Since the pretreatment with DOACs or VKA with an INR > 1.7 was contraindicated for the administration of IVT, the use of anticoagulants was either excluded or only a few anticoagulated patients were included in recent thrombectomy trials.^9^ The challenging decision of whether to conduct EVT in anticoagulated patients has been investigated in some observational studies with small sample sizes. Most of these previous studies suggested that the risk of sICH was not increased in EVT‐treated patients with prior OAC use.^1^, ^2^, ^3^, ^4^ On the contrary, some other studies observed that OAC with VKA, but not with DOACs, was an independent predictor of sICH and mortality for anticoagulated patients after EVT.^5^, ^6^, ^7^ A previous meta‐analysis showed that the anticoagulated patients seemed to be safe after EVT but with a relatively lower rate of good outcomes.^10^ The discrepancy may be due to the small sample size in these observational studies, the varied degrees of anticoagulation control, and different definitions of sICH and IVT withheld in some studies. In this study, the authors found prior VKA use within the preceding 7 days did not increase the risk of sICH overall. However, recent VKA use with an INR >1.7 was associated with a significantly increased risk of sICH compared with the non‐VKA patients.^8^ These findings provide practical information to select appropriate therapeutic strategies for AIS patients pretreated with VKA and offer access to EVT therapy for more anticoagulated patients.

Nevertheless, there are some concerns that need further elucidation. First, the present findings were derived from non‐randomized, retrospective, observational analyses, and some potential confoundings may still exist even after adjustment. For example, the histologic or imaging parameter of thrombi, the indication for EVT, the technical factors during EVT procedure, and the recanalization status were not considered. Second, the analysis was restricted to patients taking VKA within the preceding 7 days. The exact time of the last VKA intake is unknown. Third, the anticoagulated patients with DOAC were not included, which prevents the current finding from being expanded to all anticoagulated patients. Furthermore, many drugs such as aspirin, and antibiotics can interact with VKA and change the value of INR. Whether this affects the safety of EVT in VKA patients remains unknown. In this study, the authors investigated the medications prior to stroke (antiplatelets, antihypertensives, lipid‐lowering agents, and diabetes medications), and did not find these medications affect the primary endpoints, which was consistent with the previous studies.^7^ However, whether the other medications such as antibiotics were used prior to stroke in the VKA users were not referred. Finally, the patients in this study were all from American hospitals, and none of them were enrolled from Asian or European centers, which further limits the results extrapolated to other countries. Therefore, future large‐scale randomized controlled trials that incorporate clinical parameters, peripheral and imaging biomarkers, as well as technical factors influencing the recanalization of EVT are warranted to further clarify the effects and feasibility of EVT therapy in AIS patients under anticoagulation.

## FUNDING INFORMATION

L.G. is supported by the National Natural Science Foundation of China (NSFC, 81801298), Fundamental Research Funds for the Central Universities (YG2023QNB10). P.L. is supported by the National Natural Science Foundation of China (NSFC, 91957111, 81971096, 82061130224, U22A20295, and M‐0671), New Frontier Technology Joint Research sponsored by Shanghai Shenkang Hospital Development Center (SHDC12019102), Shanghai Municipal Education Commission‐Gaofeng Clinical Medical Grant Support (20181805), “Shuguang Program” supported by Shanghai Education Development Foundation and Shanghai Municipal Education Commission (20SG17), and “Shanghai Outstanding Academic Leaders Program” from Shanghai Municipal Science and Technology Committee (20XD1422400), Newton Advanced Fellowship grant provided by the UK Academy of Medical Sciences (NAF/R11/1010) and the Innovative Research Team of High‐level Local Universities in Shanghai (SHSMU‐ZLCX20211602).

## CONFLICT OF INTEREST STATEMENT

The authors declared no potential conflicts of interest with respect to the authorship, and/or publication of this article. Peiying Li is an Editorial Board member of CNS Neuroscience and Therapeutics and a co‐author of this article. To minimize bias, they were excluded from all editorial decision‐making related to the acceptance of this article for publication.

## Data Availability

Data sharing not applicable to this article as no datasets were generated or analysed during the current study.
